# Hospital Malnutrition: Prevalence, Identification and Impact on Patients and the Healthcare System

**DOI:** 10.3390/ijerph8020514

**Published:** 2011-02-16

**Authors:** Lisa A. Barker, Belinda S. Gout, Timothy C. Crowe

**Affiliations:** 1 Nutrition Department, The Royal Melbourne Hospital, Grattan Str., Parkville 3050, Victoria, Australia; E-Mail: Belinda.Gout@mh.org.au; 2 School of Exercise and Nutrition Sciences, Deakin University, Burwood Hwy, Burwood 3125, Victoria, Australia; E-Mail: tim.crowe@deakin.edu.au

**Keywords:** diagnosis-related groups, economics, hospital, malnutrition, nutrition assessment, screening

## Abstract

Malnutrition is a debilitating and highly prevalent condition in the acute hospital setting, with Australian and international studies reporting rates of approximately 40%. Malnutrition is associated with many adverse outcomes including depression of the immune system, impaired wound healing, muscle wasting, longer lengths of hospital stay, higher treatment costs and increased mortality. Referral rates for dietetic assessment and treatment of malnourished patients have proven to be suboptimal, thereby increasing the likelihood of developing such aforementioned complications. Nutrition risk screening using a validated tool is a simple technique to rapidly identify patients at risk of malnutrition, and provides a basis for prompt dietetic referrals. In Australia, nutrition screening upon hospital admission is not mandatory, which is of concern knowing that malnutrition remains under-reported and often poorly documented. Unidentified malnutrition not only heightens the risk of adverse complications for patients, but can potentially result in foregone reimbursements to the hospital through casemix-based funding schemes. It is strongly recommended that mandatory nutrition screening be widely adopted in line with published best-practice guidelines to effectively target and reduce the incidence of hospital malnutrition.

## 1. What Is Malnutrition?

Malnutrition is a broad term that can be used to describe any imbalance in nutrition; from over-nutrition often seen in the developed world, to under-nutrition seen in many developing countries, but also in hospitals and residential care facilities in developed nations. Malnutrition can develop as a consequence of deficiency in dietary intake, increased requirements associated with a disease state, from complications of an underlying illness such as poor absorption and excessive nutrient losses, or from a combination of these aforementioned factors [1,2]. Malnutrition is associated with negative outcomes for patients, including higher infection and complication rates [3–6], increased muscle loss [6–8], impaired wound healing [4,9], longer length of hospital stay [10–12] and increased morbidity and mortality [13–17].

Recently, the definition of malnutrition has been clarified by the European Society of Parenteral and Enteral Nutrition (ESPEN) to highlight the differences between cachexia, sarcopenia (loss of muscle mass and function) and malnutrition [18]. Cachexia can be defined as a “multifactorial syndrome characterized by severe body weight, fat and muscle loss and increased protein catabolism due to underlying disease(s)” [18]. Therefore, malnutrition seen in hospitalised patients is often a combination of cachexia (disease-related) and malnutrition (inadequate consumption of nutrients) as opposed to malnutrition alone. Within the context of this review, the definition of malnutrition adopted refers to the complex interplay between underlying disease, disease-related metabolic alterations and the reduced availability of nutrients (because of reduced intake, impaired absorption and/or increased losses or a combination of these) which is a combination of cachexia and malnutrition [18].

In 1859, Florence Nightingale wrote about hospitalised soldiers during the Crimea war, starving amongst plenty of food [19]. Over 100 years later, beginning from the 1970s, numerous authors have reported malnutrition rates in hospital patients to be approximately 35%, with 30 to 55% of patients entering acute hospitals being at risk of malnutrition [20–24]. Studies have also reported on factors which contribute to malnutrition (see Table 1), consequences of malnutrition and the benefit nutrition support can offer malnourished patients [15,25–27].

Over the last 30 years, advances in medical, surgical, nursing and nutrition support have been made; however, numerous publications continue to report the high prevalence and lack of awareness of malnutrition [11,17,28]. To improve detection rate, many studies have investigated various methods for screening and assessing malnutrition, and shown many of these tools to be simple, fast, accurate and of utility in the clinical setting [29–33]. Despite the availability of such tools, malnutrition prevalence remains high and appropriate treatment is not always being delivered. This review aims to briefly summarise nutrition screening and assessment tools available for use in hospitalised patients and provides a short discussion on malnutrition prevalence, consequences and treatment to provide clinicians with a starting point for achieving successful malnutrition identification.

## 2. Malnutrition Screening and Assessment

Identifying malnutrition or malnutrition risk is fundamental to its treatment. It is therefore unsurprising that many validated tools for nutrition risk screening and nutrition assessment exist for the clinician to use in assisting with the accurate identification, referral and treatment of patients who are malnourished or at risk of malnutrition.

The American Dietetic Association defines nutrition risk screening as “the process of identifying patients with characteristics commonly associated with nutritional problems who may require comprehensive nutrition assessment” [34]. In straightforward terms, nutrition risk screening refers to a rapid and simple set of usually two or three questions that have been validated to predict malnutrition risk. Patients identified through screening as ‘at risk’ are subsequently referred for further nutritional assessment, usually performed by a dietitian. Nutrition screening can be performed by any trained health professional, but is usually completed by nursing or nutrition assistant staff.

In contrast to nutrition risk screening, the American Dietetic Association defines nutrition assessment as “a comprehensive approach to defining nutritional status using medical, nutritional, and medication histories; physical examination, anthropometric measurements and laboratory data” [34]. Essentially, nutrition assessment is a diagnostic tool to determine if a patient is currently malnourished, though does require greater skill and time to perform than nutrition risk screening.

Patients are generally referred to a dietitian by medical and nursing staff to provide nutritional care, and little time often exists for these staff to screen additional patients [35]. It is therefore of concern that many malnourished patients in acute settings are not identified as such, and thereby not referred for nutrition assessment and treatment. In some countries, namely the United Kingdom, United States, the Netherlands and some parts of Denmark, nutrition screening on patient admission is mandatory, with satisfactory hospital accreditation dependent on this being carried out [30]. In Australia however, this is currently not the case. A seminal study in 1998 looking at screening practices of dietitians in Australian hospitals surveyed dietitians on their usual practice and perceived barriers to nutrition risk screening. Of alarm, only 5% of 124 hospitals whose dietitians participated in the survey carried out routine nutrition risk screening, as required by hospital policy [35]. This clearly shows nutrition risk screening requires an all-of-hospital approach to prevent movement down the list of patient management priorities.

### Nutrition Screening and Assessment Tools

Numerous nutrition screening and assessment tools exist to identify risk of, and diagnose, malnutrition. Recent evidence-based practice guidelines published by the Dietitians Association of Australia considered levels of evidence for use of validated screening and assessment tools in the acute setting as well as other areas [36]. They reported on five screening and three assessment tools validated for use in the acute setting. These guidelines provided a Grade B (Good) National Health and Medical Research Council (NHMRC) grade of recommendation that routine nutrition risk screening should occur within the acute setting, but only a Grade D (Poor) recommendation for the adoption of routine screening in the sub-acute, residential aged care and community setting. A detailed description highlighting the developmental background and validation process for each of the discussed tools which formed part of the Guideline development is beyond the scope of this review; however, a brief description of the main tools outlining their strengths and weaknesses is included following.

The Malnutrition Screening tool (MST) is a simple, three-question tool assessing recent weight and appetite loss validated for use in general medical, surgical and oncology patients [37,38]. It was designed for use by non-nutrition-trained staff and utilises a scoring system to identify patients at high nutrition risk which can then provide a basis for dietetic referrals and intervention [37,39]. Related to the MST, the Malnutrition Universal Screening Tool (MUST) was developed to detect both under-nutrition and obesity in adults, and was designed for use in multiple settings including hospitals and nursing homes. Body Mass Index (BMI), unplanned weight loss and the presence or absence of serious disease allow a score to be derived to indicate whether nutrition intervention is necessary. The MUST has been determined to consistently give reliable results; however, is limited by the fact it has not been validated in children or renal patients [40–43].

The Mini Nutrition Assessment (MNA) was developed specifically for use among elderly patients (≥65 years) in hospitals, nursing homes and the community and is thus limited to this demographic [37,44]. The original form considers anthropometrical, medical, lifestyle, dietary and psychosocial factors in an 18 item assessment, using a points-based scoring system to determine if a patient is at risk of, or suffering from, malnutrition[44,45]. The short-form MNA (MNA-SF), which is an abridged version of the MNA, provides a simple two-step nutrition screen with the full MNA completed only for those patients deemed at nutritional risk [46–48].

Nutritional Risk Screening (NRS-2002) uses recent weight loss, decreased BMI and reduced dietary intake, combined with a subjective assessment of disease severity (based on increased nutrition requirements and/or metabolic stress), to generate a nutrition risk score [37]. Such subjective grading of illness severity may not accurately reflect current nutritional status and the tool does not allow for definitive diagnosis of malnutrition. The NRS tool has, however, been recommended for use in hospitalised patients by ESPEN and may be useful for prompting the initiation of nutrition support [41,49].

The four item Short Nutrition Assessment Questionnaire (SNAQ) was developed to diagnose malnutrition in hospitalised patients and provides an indication for dietetic referrals as well as outlining a nutrition treatment plan [37,50]. It has been validated for hospital inpatient and outpatient use, as well as residential patients and does not require calculation of BMI [51,52].

Subjective Global Assessment (SGA) is one of the most commonly used nutrition assessment tools, and assesses nutrition status via completion of a questionnaire which includes data on weight change, dietary intake change, gastrointestinal symptoms, changes in functional capacity in relation to malnutrition as well as assessment of fat and muscle stores and the presence of oedema and ascites [53]. This tool allows for malnutrition diagnosis, and classifies patients as either: A—well-nourished; B—mildly/moderately malnourished; or C—severely malnourished.

SGA has been found to be an appealing method of assessing nutritional status, as its subjective nature allows clinicians to capture subtle patterns of change in clinical variables (e.g., weight loss patterns rather than absolute weight loss). A high degree of inter-rater reproducibility has been shown for SGA, with 91% of surgical patients classified by SGA having two clinicians agreeing on SGA classification [53].

It is, however, important to note that the previously mentioned screening tools do not allow a specific diagnosis of malnutrition versus cachexia, and that in fact, many patients diagnosed as at risk of, or actually malnourished, may be better classified by the definition of malnutrition used in this review (incorporating cachexia). As it stands, there are no specific screening tools to identify cachexia versus malnutrition, however some questionnaires exist to diagnose anorexia/cachexia in cancer patients, and readers are referred to the consensus opinion paper discussing this issue [18].

## 3. Malnutrition Prevalence in the Acute Setting

Malnutrition prevalence in the hospital (acute) setting has been widely documented in the literature to be between 20% and 50%, depending on the patient population and definition and criteria used for diagnosis [1,54,55]. Rather than only reporting on malnutrition rates, many of these aforementioned malnutrition prevalence studies have also considered other aspects of patient care affected by malnutrition, namely length of stay (LOS), medication use, infection rates, dietetic referrals, documentation of malnutrition and mortality.

An Australian study in 2009 [11] reported that in a Melbourne tertiary teaching hospital, malnutrition was identified in 23% of 275 patients randomly assessed by SGA on admission. The authors found malnourished patients had a significantly longer LOS by 4.5 days compared to well-nourished patients. Of concern from this study considering the high rate of malnutrition identified, a dietitian was only referred to 36% of these patients during their admission, and only 7 out of 24 cases (29%) were correctly documented in the medical history as such by the dietitian.

Further Australian studies showed similar results to the study previously described. In 2007, SGA was used to assess the nutritional status of patients in a private hospital and reported a malnutrition rate of 42%, with only 15% of these patients referred to a dietitian [56]. In the setting of a public teaching hospital, malnutrition rates and awareness of malnutrition and its risk factors by medical and nursing professionals in elderly patients was assessed [57]. The study reported a prevalence rate for malnutrition of 30% using MNA. Documentation of weight and appetite loss by medical and nursing staff was found to be poor at just 19% and 53% respectively, with dietetic referrals being made for only 7% and 9% of these patients respectively.

In 2002 and 2003, twenty Queensland public acute-care facilities conducted a single-day audit of nutritional status using SGA and found malnutrition rates to be 35% and 31% respectively [55]. Also documented as part of the two audits were variables found to be significantly associated with an increased risk of malnutrition, namely older age, metropolitan location of facility and medical specialty.

Middleton and colleagues also used the SGA tool to assess malnutrition rates in a metropolitan teaching hospital setting and found a malnutrition rate of 36% in the two hospitals studied [17]. Twelve-month follow-up of patients assessed as malnourished found a longer LOS (17 days *versus* 11 days for well-nourished patients) and higher mortality rates of 30% compared to 10% for well-nourished patients.

European, American and South American data in comparable settings reflect similar malnutrition rates to Australia. A German study published in 2006 reported a 27% rate of malnutrition (using SGA), with malnourished patients having a LOS 43% longer than well-nourished patients [54]. A Danish group used NRS to determine nutrition risk and found 40% of patients to be as such, with only 8% documented as malnourished [58]. Two English studies in 2000 and 2003 reported malnutrition rates of 20% and 19% respectively, with the former study reporting malnourished patients having a longer LOS by 3 days and higher rates of medical prescriptions and infections compared to well-nourished patients [28,59]. Two further studies using SGA reported prevalence rates of malnutrition of 48% and 45% with again poor medical documentation and longer LOS in malnourished patients [1,60].

It is clear from the number of published studies; malnutrition is a worldwide problem with poor diagnosis and documentation rates and higher LOS and infection rates commonly reported.

## 4. Malnutrition and Its Associated Consequences

Malnutrition has often been referred to as the “skeleton in the hospital closet”, as it is often overlooked, undiagnosed and untreated [61,62]. Despite this, the negative consequences of malnutrition have been widely reported in the literature, and can be separated into two main categories: consequences for the patient and consequences for the health care facility.

### 4.1. Consequences for the Patient

Malnutrition has been shown to cause impairment at a cellular, physical and psychological level [14–16]. This impairment is dependent on many factors, including the patient’s age, gender, type and duration of illness, and current nutritional intake. On a cellular level, malnutrition impairs the body’s ability to mount an effective immune response in the face of infection, often making infection harder to detect and treat [63]. It also increases the risk of pressure ulcers, delays wound healing, increases infection risk, decreases nutrient intestinal absorption, alters thermoregulation and compromises renal function [1,14–16].

On a physical level, malnutrition can cause a loss of muscle and fat mass, reduced respiratory muscle and cardiac function, and atrophy of visceral organs [6,14,15]. It has been shown that an unintentional 15% loss of body weight causes steep reductions in muscle strength and respiratory function, while a 23% loss of body weight is associated with a 70% decrease in physical fitness, 30% decrease in muscle strength and a 30% rise in depression [16]. At a psychological level, malnutrition is associated with fatigue and apathy, which in turn delays recovery, exacerbates anorexia and increases convalescence time [15].

It is widely reported in the literature that malnutrition is associated with an increased length in hospital stay [10,64,65]. One study conducted in the United States looked at adult patients hospitalised for more than 7 days and examined the impact nutritional decline had on outcomes, including LOS [10]. The results showed that patients who were admitted with some degree of malnutrition, and those patients who experienced a decline in nutritional status during their admission, had significantly longer hospital stays (by an average of 4 days) than patients both admitted and discharged as well nourished. Similarly, a study conducted in Australia found a significantly greater difference of 5 days between the LOS of malnourished and well-nourished patients [17].

In addition to a longer LOS, malnourished patients are more prone to experiencing complications during their period of hospitalisation than patients who are in a well-nourished state [10]. Complications can occur when an unexpected accident or disease adds to a pre-existing illness without being specifically related to the illness [1]. For example, one study that assessed the nutritional status of patients preoperatively found that malnourished patients had significantly higher rates of both infectious and non-infectious complications [66]. Following on from a higher complication risk, as mentioned prior malnutrition has also been shown to be associated with an increase in mortality rates [16,17,67].

Despite the multitude of evidence indicating that patients who are nutritionally compromised suffer worse outcomes, it is difficult to control for disease severity in the clinical setting and thus definitively conclude that malnutrition alone is a cause of these outcomes. The fact that numerous studies internationally, in a wide variety of clinical settings and patient groups, all report similar findings lends strength to the premise that malnutrition is detrimental in terms of clinical outcome. The high prevalence rates of malnutrition in the hospital setting indicate that such negative outcomes as longer hospital stay, higher complication and infection rates, and mortality would be highly prevalent also. It is therefore not surprising that malnutrition has significant secondary effects to health care facilities.

### 4.2. Consequences for the Health Care Facility

Malnutrition places additional stress on acute health care facilities. As previously stated, malnourished patients often have higher rates of infections and pressure ulcers (and consequently require greater nursing care), require more medications, are less independent due to muscle loss and consequently have longer lengths of hospital stay [17,54,60,68]. All these issues combined indirectly increase hospital costs associated with treating the patient, secondary to the management of their primary medical reason for admission.

Malnutrition also has an indirect effect on health care costs by way of the casemix funding system, as exists in much of Australia and other countries around the world. Under casemix-based funding, once a patient is discharged, their medical notes are audited by medical coders and their major diagnosis, surgeries, co-morbidities, complications and other interventions are recorded and a Diagnosis Related Group (DRG) is assigned. In Australia at least, hospitals are subsequently reimbursed for the patient admission based on the DRG. Malnutrition, when documented as a co-morbidity or complication, has the ability to influence a DRG, often resulting in a “higher’ classification which has the potential to attract greater hospital reimbursement [69].

Two Australian studies have reported estimates of unclaimed reimbursements from patient admissions where malnutrition was not recorded as a co-morbidity as part of the DRG. In Melbourne in 2009, a study used SGA to diagnose malnutrition across a large hospital-based population and estimated an annualised deficit to the hospital in reimbursements of AUD1,850,540 for undiagnosed or undocumented cases of malnutrition [11]. Similarly, a study conducted in Brisbane also used SGA to determine nutrition status in a hospital population and estimated an annualised financial loss to the hospital of AUD1,677,235 due to undiagnosed and documented malnutrition [70].

Two international studies, one in Germany and one in the United States, both reported financial losses to the hospital due to unrecognised malnutrition based on a DRG funding system. In the German study, SGA was used to define malnutrition and reported a 19% rate of malnutrition in the patient population, an increased LOS of 4 days in the malnourished patient group, and an annual financial shortfall of €35,280 due to unrecognised malnutrition [71]. Similarly, the American study reported a loss of greater than USD86,000 after conducting a retrospective audit of patient medical charts [68].

Other studies have also shown increased financial costs to health care facilities due to untreated malnutrition based simply on the increased LOS associated with malnutrition. To illustrate, one study found that patients at risk of malnutrition had a 6-day longer LOS than those not at nutritional risk, resulting in treatment costs for malnourished patients increasing by USD1,633 per patient per hospital stay [72]. Taking the opposite approach, a similar study looked at the cost-benefit associated with nutritional intervention in patients at risk of malnutrition and found that early intervention using specialised nutritional products and frequent reviews was more cost-effective than either early intervention or frequent review alone, with an estimated saving to the health care facility of USD1,064 per patient [73].

It seems evident by the number of researchers who have examined the relationship between malnutrition and its effect on both patients and treatment costs, that benefits to both individuals and hospitals exist if malnutrition is correctly identified and treated.

## 5. Malnutrition Identification and Treatment

In order to prevent or reverse the associated negative clinical outcomes for malnourished patients, it is imperative that these patients are promptly identified. Routine nutrition screening using one of the previously described validated nutrition screening tools can provide a basis for dietetic referrals for prescription of appropriate nutrition support.

A Cochrane review published in 2008 examined evidence surrounding dietary advice and the nutritional intake of adults with illness-related malnutrition [74]. The review comprised 36 studies with 2,714 participants and compared a combination of dietary advice, dietary supplements or no advice with outcome measures including mortality, morbidity, weight, nutrient intake and measures of clinical function. The authors concluded that dietary advice plus nutritional supplements may be more effective than advice alone or no advice on the measure of short-term weight gain, but highlighted the lack of evidence in the management of illness-related malnutrition. The lack of an unequivocal finding of the benefit of nutritional intervention in malnourished patients is likely related to the difficulties in performing high-quality randomised-controlled trials owing to the ethical concerns of withholding nutrition support to patients identified as malnourished.

Some smaller studies have reported positive results with nutritional intervention in malnutrition including a survey of 19 United States hospitals which demonstrated that hospital LOS was influenced by the degree of nutritional care received by patients at risk of malnutrition [73]. Patients who received high quality nutritional care (defined as early intervention plus frequent use of nutrition services) averaged a 2.2 day shorter period of hospitalisation than those who received medium quality nutritional care (defined as early intervention OR frequent use of nutrition services). Those who received low quality nutritional care (defined as late or no intervention and/or infrequent or no use of nutrition services) had the longest average period of hospitalisation; however, the notion of low quality care does raise ethical concerns.

An Australian study conducted in 2005 investigated the impact of early and intensive nutrition intervention among outpatients receiving radiotherapy to the gastrointestinal or head and neck area [75]. Those patients who received individualised nutrition intervention were significantly more weight stable and experienced significantly less deterioration in nutritional status than patients who received usual nutrition care. Several other randomised controlled trials looking at nutrition advice and supplementation, as opposed to supplementation alone, on outcomes such as body weight, quality of life, muscle function and hospital readmission show favourable results for a combination of dietary counselling and supplementation [76–79].

With the substantial body of evidence highlighting the clinical risks that malnutrition poses to patients and the benefits that nutrition advice and supplementation can have, it is clear that nutrition screening allows for early identification and treatment of malnutrition, thus optimising the patient’s chances of attenuating some or all of the adverse outcomes associated with malnutrition

## 6. Conclusions and Recommendations

Malnutrition is prevalent around the world and is a burden on patients and health care facilities. Despite numerous advances in medicine and clinical care, the simple correction of a patient’s nutritional status appears to be overlooked or not considered as a sufficient medical priority.

The treatment of malnutrition first requires a malnourished patient to be identified via either screening or assessment. This needs to be done on admission, and preferably made mandatory by health care accrediting bodies. In order to achieve this, dietitians need to have the confidence and knowledge to detect malnutrition, which ideally will be done using a validated assessment tool such as SGA for example. In order for patient outcomes and financial benefits to be monitored, adequate documentation of malnutrition is essential.

In Australia, the recently published Dietitians Association of Australia Best Practise Guidelines further strengthens the argument for the implementation of routine nutrition screening, especially in the acute hospital setting where substantial evidence indicates the high burden of malnutrition. Screening alone is, however, only one part of the solution. A clear nutrition care pathway should indicate the action required based on the screening result. As screening will inevitably create an increased number of dietetic referrals to assess these potentially malnourished patients additional funding would allow for extra staff to deal with the increased dietetic workload.

## Figures and Tables

**Table 1.** Factors contributing to malnutrition in acute care patients (reproduced with permission from [15]; published by Elsevier, 2007).

**Personal**	**Organisational**
Age	Failure to recognise malnutrition
Apathy/depression	Lack of nutritional screening or assessment
Disease (e.g., cancer, diabetes, cardiac, gastrointestinal)	Lack of nutritional training
Inability to buy, cook or consume food	Confusion regarding nutritional responsibility
Inability to chew or swallow	Failure to record height and weight
Limited mobility	Failure to record patient intake
Sensory loss (taste, smell)	Lack of adequate intake
Treatment (ventilation, surgery, drain tubes)	Lack of staff to assist with feeding
Drug therapy	Importance of nutrition unrecognised
